# MERTK missense variants in three patients with retinitis pigmentosa

**DOI:** 10.1080/13816810.2022.2113541

**Published:** 2022-08-29

**Authors:** Federica E. Poli, Imran H. Yusuf, Penny Clouston, Morag Shanks, Jennifer Whitfield, Peter Charbel Issa, Robert E. MacLaren

**Affiliations:** a Nuffield Laboratory of Ophthalmology, Nuffield Department of Clinical Neurosciences, University of Oxford, Oxford, UK; b Oxford Eye Hospital, Oxford University Hospitals NHS Foundation Trust, Oxford, UK; c Oxford Regional Genetics Laboratories, Oxford University Hospitals NHS Foundation Trust, Oxford, UK

**Keywords:** Retinitis pigmentosa, MERTK, mutation, gene therapy, CRISPR-Cas

## Abstract

**Background:**

*MERTK (MER proto-oncogene, tyrosine kinase)* is a transmembrane protein essential in regulating photoreceptor outer segment phagocytosis. Biallelic mutations in *MERTK* cause retinal degeneration. Here we present the retinal phenotype of three patients with missense variants in *MERTK*.

**Materials and methods:**

All patients underwent a full clinical examination, fundus photography, short-wavelength fundus autofluorescence and optical coherence tomography imaging. Two patients also underwent Goldmann visual field testing and electroretinography was undertaken for the third patient. Molecular genetic testing was undertaken using next generation or whole-exome sequencing with all variants confirmed by Sanger sequencing.

**Results:**

The first patient was a 29-year-old female heterozygous for a missense variant (c.1133C>T, p.Thr378 Met) and a nonsense variant (c.1744_1751delinsT, p.Ile582Ter) in *MERTK*. The second patient was a 26-year-old male homozygous for a c.2163T>A, p.His721Gln variant in *MERTK*. The third patient was an 11-year-old female heterozygous for a deletion of exons 5–19 and a missense variant (c.1866 G>C, p.Lys622Asn) in *MERTK*. Reduced night vision was the initial symptom in all patients. Fundoscopy revealed typical signs of retinitis pigmentosa (RP) with early-onset macular atrophy. All three *MERTK* missense variants affect highly conserved residues within functional domains, have low population frequencies and are predicted to be pathogenic *in silico*.

**Conclusions:**

We report three missense variants in *MERTK* and present the associated phenotypic data, which are supportive of non-syndromic RP. *MERTK* is a promising candidate for viral-mediated gene replacement therapy. Moreover, one variant represents a single nucleotide transition, which is theoretically targetable with CRISPR-Cas9 base-editing.

## Introduction

Retinitis pigmentosa (RP) is a set of clinically and genetically heterogenous inherited retinal dystrophies characterised by progressive primary rod photoreceptor degeneration with concurrent, or later degeneration of cones (1). It typically manifests with difficulties in dark adaptation and night vision, followed by progressive peripheral visual field loss, with subsequent loss of central vision. A vast number of heterogenous genetic defects have been implicated in the pathogenesis of non-syndromic RP (2,3).

**Figure 1.**
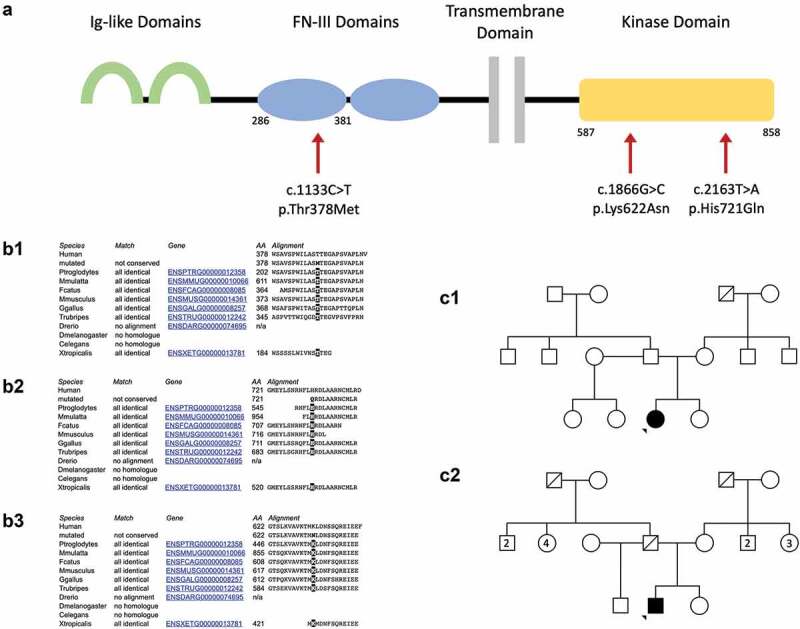
Panel A: structure of the MERTK transmembrane protein. The extracellular portion includes two immunoglobulin-like (Ig-like) domains (green) and two fibronectin type III (FN-III) domains (blue). The intracellular region contains a highly conserved kinase domain (yellow). The location of the Three mutations discussed are indicated by red arrows. The respective amino acid residues corresponding to the domains affected are indicated below the protein schematic. Panel B: Conservation across species of the amino acid residue subject to mutation in the c.1133c>t, p.Thr378 met variant (B1), the c.2163t>a, p.His721gln variant (B2) and the c.1866G>C, p.Lys622Asn variant (B3). Panel C: Pedigrees for Case 1 (C1) and Case 2 (C2). Pedigree not available for Case 3. Note the pedigrees are based on clinical presentation and segregation has not been possible.

The MER proto-oncogene, tyrosine kinase (*MERTK*) gene encodes a transmembrane protein expressed in the retinal pigment epithelium (RPE), which plays a critical role in photoreceptor homeostasis by regulation of phagocytosis of shed photoreceptor outer segment discs (Figure 1, panel A) (4,5). Numerous mutations in *MERTK* have been identified as pathogenic for retinal dystrophies (6,7). *MERTK* mutations cause a rod-cone dystrophy with early macular atrophy, with RP being the most common retinal phenotype, although cases of Leber congenital amaurosis have been reported (7).

Herein, we describe three patients who presented with an autosomal recessive RP phenotype and were identified to have biallelic *MERTK* mutations. These missense variants in *MERTK* have been previously reported in isolation without detailed phenotypic data (7–10). Reporting rare missense mutations and describing the associated phenotype builds the evidence base for the pathogenicity of a given variant, identifies exons that are essential for retinal function and identifies variants that are targetable by currently available gene-editing tools.

## Materials and methods

The three patients were referred to the Oxford Eye Hospital, a tertiary referral centre for inherited retinal degenerations. Data were collected retrospectively using clinical information and investigations undertaken as part of their routine clinical care. These included best-corrected visual acuity, Goldmann kinetic visual field testing, slit lamp and fundoscopic examination. Retinal images included digital fundus photography (Optos, Dunfermline, Scotland); short-wavelength fundus autofluorescence (FAF) with optical coherence tomography (OCT) imaging (Heidelberg Spectralis, Heidelberg Engineering GmbH, Heidelberg, Germany). Full-field electroretinography was undertaken according to ISCEV standards for Case 3.

Molecular genetic testing was undertaken as previously described (11). For Cases 1 and 2, a panel of 111 genes associated with RP or RP-like phenotypes was utilised for genetic characterisation. A customised HaloPlex Target Enrichment system (Agilent Technologies, Santa Clara, United States) was used, and sequencing was done with the Illumina MiSeq technology. For Case 3, TWIST Singleton whole-exome sequencing with targeted analysis of a panel of 199 genes associated with R32 Retinal disorders was used. The Twist Human Core Exome Multiplex Hybridization Kit (Twist Bioscience, San Francisco, United States) was used as the method of enrichment and the Illumina NovaSeq6000 technology was used for sequencing. Both sequencing technologies involve data generated by the High-Throughput Genomics Group at the Wellcome Trust Centre for Human Genetics, Oxford. Data were analysed using a validated in-house analysis pipeline. All variants identified were confirmed by Sanger sequencing. For Case 3, SNP microarray was used to confirm the large deletion. SNP microarray processing was carried out using the Illumina GSAv3 microarray (>665,000 probes, mean OMIM gene resolution 15kb minimum). Analysis and interpretation were carried out using NxClinical 6.1 (BioDiscovery) and the FASST2 CNV calling algorithm on human genome build GRCh37(hg19).

## Results

### Case 1

A 29-year-old female was referred to our clinic for assessment of RP which was diagnosed 6 years earlier. She reported progressive nyctalopia from childhood with worsening daytime vision and glare. No other family member had a history of RP (Figure 1, panel C1) and there was no known consanguinity in her family who was originally from Cyprus. Ophthalmic assessment performed abroad as a young child did not detect any abnormalities.

**Figure 2.**
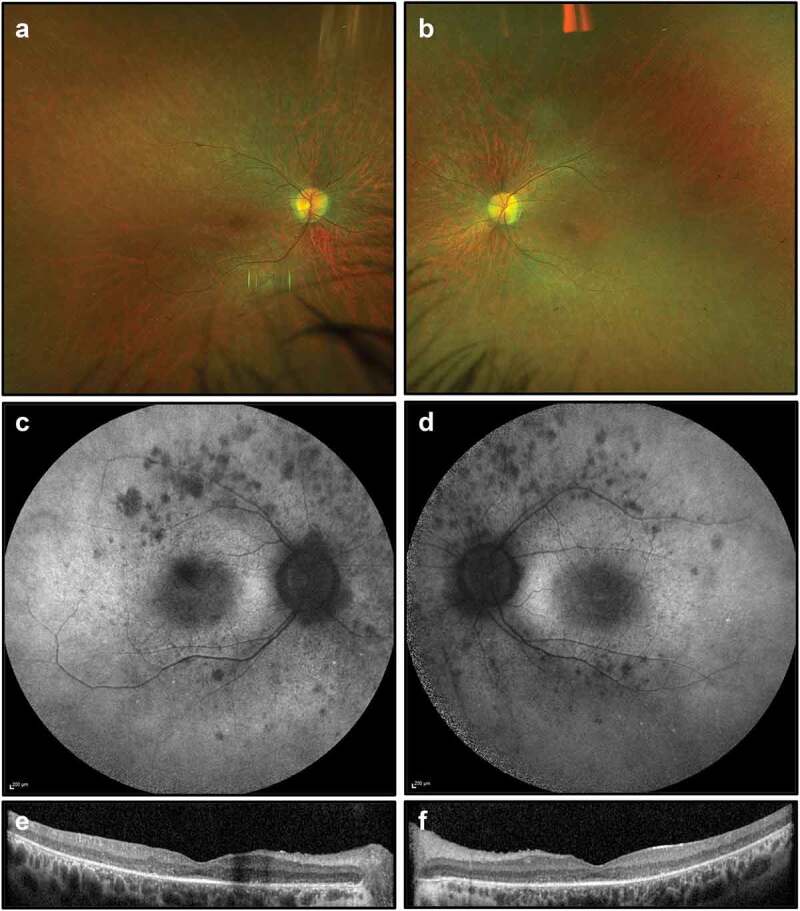
Retinal imaging studies for Case 1: (a, b) colour photographs of right and left fundi demonstrating attenuated vessels and waxy disc pallor but not capturing sparse bone spicule pigmentations; (c, d) widefield FAF imaging (55 degrees) demonstrating patchy autofluorescence in the nasal midperiphery and along the supero-temporal arcades; (e, f) OCT imaging showing extensive photoreceptor loss and outer retinal degeneration, with a partially preserved foveal ellipsoid zone.

**Figure 3.**
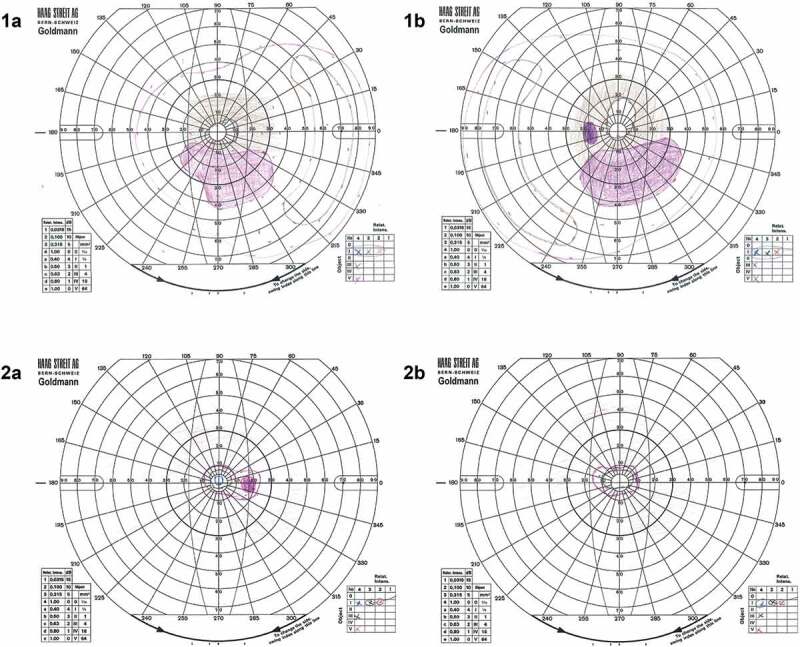
Goldmann visual fields for Case 1 (1a) right and (1b) left eyes showing a bilateral residual temporal island as is often seen in advanced RP with a bilateral inferior Scotoma that matches the pattern of degeneration on FAF imaging in Figure 2. Case 2 (2a) right and (2b) left demonstrates generalised, severe visual field constriction with the V4 isopter constricted to approximately 10 degrees in both eyes.

On examination, best corrected visual acuity was 6/24 in the right eye and 6/36 in the left eye. She had normal anterior segments with no cataract. There were a few vitreous cells bilaterally. Retinal examination demonstrated bilateral attenuated vessels and sparse bone spicule pigmentation, consistent with RP. FAF imaging showed patchy hypoautofluorescent spots most marked in the nasal midperiphery and along the supero-temporal arcade. OCT demonstrated extensive photoreceptor loss with a partially preserved foveal ellipsoid zone (Figure 2). Goldmann visual fields were constricted bilaterally (Figure 3).

Molecular genetic testing identified a missense variant in *MERTK* (c.1133C>T, p.Thr378 Met, genomic coordinates Chr2.hg19:g. 112,733,038) and a heterozygous nonsense variant in *MERTK* (c.1744_1751delinsT, p.Ile582Ter, genomic coordinates Chr2.hg19:g. 112760722_112760728). The c.1133C>T variant affects the highly conserved amino acid residue threonine in position 378, found within the extracellular fibronectin type III functional domain (Figure 1, panel B1). It has a low population frequency in genetic databases and is predicted to be pathogenic *in silico* (Table 1, for additional details refer to Supplements). It was not possible to obtain segregation studies for this patient.

**Table 1.** Summary of the characteristics of the *MERTK* missense variants.

Case	*MERTK*Variant	Exon	Protein change	Population frequency (gnomAD)	Position	ACMG/AMP classification	Pathogenic prediction (PolyPhen2)
1	c.1133C>T	7	p.Thr378 Met	South Asian: 4 of 15,308 (0.3%)	Fibronectin type III domain (AA 286–381). Extracellular functional domain.	Uncertain pathogenicity (PM2, PP3)	Score = 1 Probably damaging
				East Asian: 2 of 9,195 (0.02%)			
				Non-Finnish European: 1 of 56,875 (0.002%)			
2	c.2163T>A	16	p.His721Gln	Non-Finnish European: 1 of 56,868 (0.002%)	Tyrosine kinase domain (AA 587–858). Intracellular highly conserved catalytic domain.	Uncertain pathogenicity (PM2, PP3)	Score = 1 Probably damaging
3	c.1866 G>C	13	p.Lys622Asn	Non-Finnish European: 1 of 56,875 (0.002%)	Tyrosine kinase domain (AA 587–858). Intracellular highly conserved catalytic domain.	Uncertain pathogenicity (PM2, PP3, PM3_Moderate)	Score = 1 Probably damaging

For further details of *in silico* predictions, refer to Supplements.

### Case 2

A 26-year-old male was seen in our clinic with gradual visual loss over the preceding 2 years. He first noticed night and colour vision problems as a teenager. His vision subsequently declined over recent years with marked glare and reading difficulties. There was no known family history of vision problems (Figure 1, panel C2). His parents were from the same region in Albania, but there was no known consanguinity. The younger sister was also examined and had a normal fundoscopic examination.

**Figure 4.**
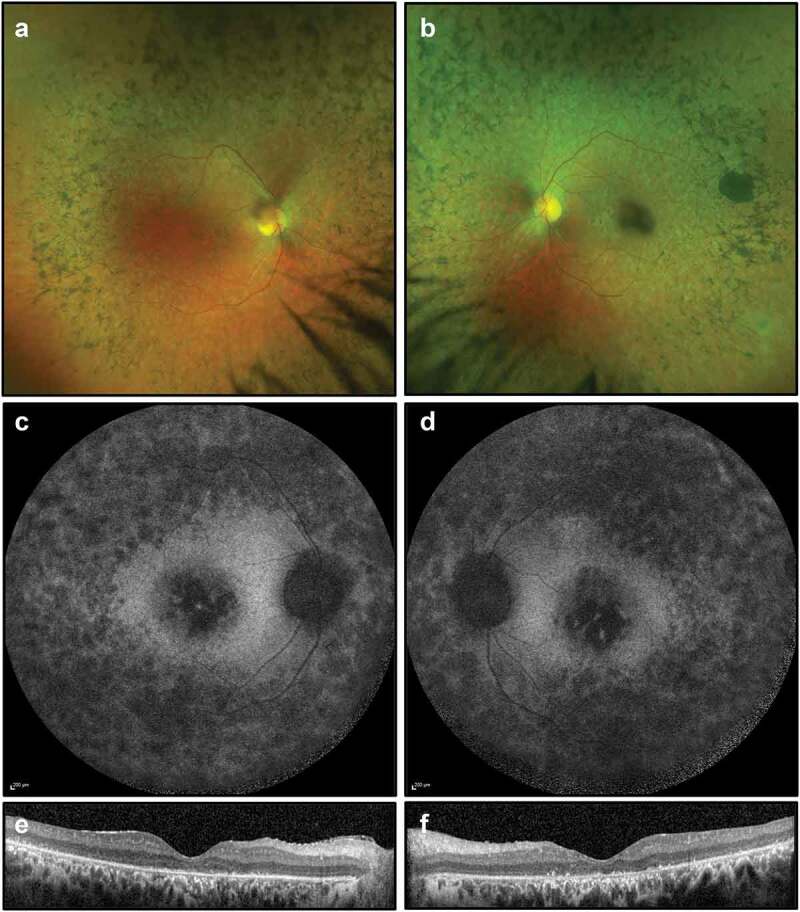
Retinal imaging for Case 2: (a, b) colour photographs of fundi demonstrating widespread peripheral bone spicule pigmentations and attenuated vessels; (c, d) widefield FAF imaging (55 degrees) demonstrating widespread autofluorescence atrophy and macular atrophy with no ring of increased autofluorescence; (e, f) OCT imaging showing extensive photoreceptor loss, outer retinal degeneration, absence of foveal ellipsoid zone and presence of subretinal material, thought to be secondary to the defective phagocytic process by the RPE.

At the time of presentation (26 years of age), best-corrected visual acuity was 6/12–1 in the right eye and 6/60 in the left eye. This deteriorated over 3 years to 6/48 in the right eye and counting fingers in the left eye. On examination, he had bilateral mild posterior subcapsular cataract, vitreous cells, retinal arteriolar constriction, atrophic retinal changes with bone spicule pigmentation and marked macular atrophy (Figure 4). FAF imaging demonstrated widespread peripheral and macular atrophy, with no ring of increased autofluorescence. OCT showed extensive photoreceptor loss, outer retinal degeneration, presence of subretinal material and absence of foveal ellipsoid zone (Figure 4). 18 months later, OCT imaging showed mild macular cystoid changes, more pronounced in the right eye. Dorzolamide drops, used three times daily, were trialled for degenerative cystoid macular oedema with minimal clinical improvement. His visual acuity, dark adaptation and visual fields deteriorated significantly to the point where he was unable to work.

Molecular genetic testing identified a homozygous variant in *MERTK* (c.2163T>A, p.His721Gln, genomic coordinates Chr2.hg19:g.112,777,073). This causes a substitution of the amino acid residue in position 721 from histidine to glutamine within the highly conserved intracellular tyrosine kinase domain (Figure 1, panel B2). It has only been identified in 1 of 56,868 Non-Finnish European individuals in genetic databases and is predicted to be pathogenic *in silico* (Table 1, for additional details refer to Supplements).

### Case 3

An 11-year-old female was referred to our clinic for specialist assessment due to parental concerns regarding poor night vision. She reported problems with low light and night vision for as long as she could remember. There was no known family history of any retinal dystrophies or eye conditions and no known consanguinity.

**Figure 5.**
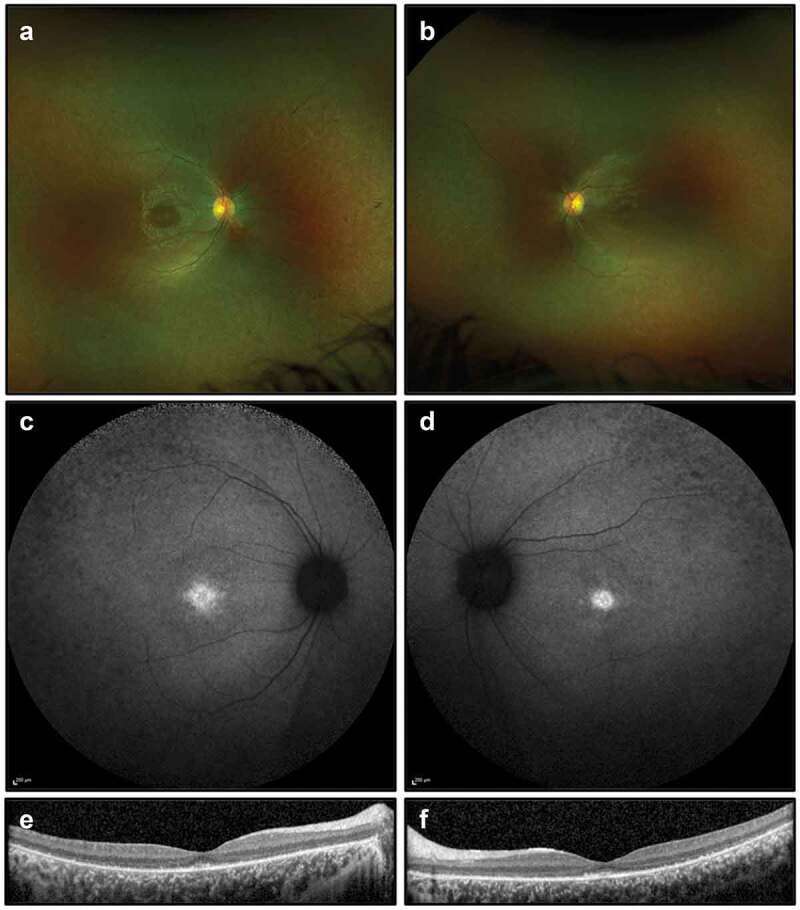
Retinal imaging for Case 3: (a, b) colour photographs of fundi demonstrating sparse peripheral pigment migration; (c, d) widefield FAF imaging (55 degrees) showing foveal hyper-autofluorescence; (e, f) OCT imaging showing outer retinal degeneration with some preservation of the foveal ellipsoid zone.

Best corrected visual acuity was 6/9 in both eyes, with no improvement on pinhole testing. On examination, she had healthy anterior segments, thin retina with pigment migration in both eyes. FAF imaging showed bilateral foveal hyper-autofluorescence and OCT demonstrated macular thinning with outer retinal degeneration (Figure 5). Full-field electroretinography demonstrated absent rod responses and severely attenuated cone responses.

Molecular genetic testing identified a pathogenic heterozygous deletion of exons 5–19 in *MERTK* (genomic coordinates Chr2.hg19:g 112,722,768– 112,786,441), confirmed using a SNP microarray. NGS dosage data suggested that the deletion event may have included exons 3 and 4; however due to probe coverage on the SNP microarray it was only possible to confirm a deletion of *MERTK* exons 5–19. It also identified a heterozygous missense variant in *MERTK* (c.1866 G>C, p.Lys622Asn, genomic coordinates Chr2.hg19:g. 112,761,560) of uncertain significance, previously reported by Ellingford et al (8). The c.1866C>G missense variant affects the highly conserved lysine amino acid residue in position 622 (Figure 1, panel A and B3). It has a low population frequency in genetic databases and is predicted to have a deleterious effect upon protein function *in silico* (Table 1, for additional details refer to Supplements). It was not possible to obtain segregation studies for the patient.

## Discussion

The *MERTK* gene is located on the long arm of chromosome 2 (2q14.1). It encodes the MER receptor tyrosine kinase, a 999 amino acid transmembrane protein that functions as a signalling molecule in the phagocytic pathway in the RPE (4). As part of the process of photoreceptor outer segment disc renewal, shed outer segments discs are phagocytosed by the RPE cells (5–7). This is a continual process essential in preventing accumulation of toxic debris in the subretinal space. The association between retinal degeneration and the *MERTK* gene was first identified in the Royal College of Surgeons rat (12,13). In this naturally occurring model of retinal dystrophy, the failure the RPE to phagocytose shed discs (14) leads to accumulation of debris and photoreceptor cell degeneration (15,16). Autosomal recessive variants in *MERTK* were subsequently identified in humans with an RP phenotype, confirming the mechanism of defective RPE-mediated phagocytosis in human retinal disease for the first time (6). To date, numerous *MERTK* variants have been reported in patients with inherited retinal dystrophies, with 79 variants identified up to 2018 (7) and further variants reported in more recent years (17–20). Inherited retinal dystrophies associated with *MERTK* mutations are characterised by early macular involvement in addition to more peripheral retinal abnormalities (7,21), and the reduced outer segment phagocytosis seems to result in reduced lipofuscin accumulation (22).

The c.1133C>T, p.Thr378 Met missense variant identified in Case 1 has only been previously reported in a homozygous 21-year-old male, without presentation of detailed clinical data (9). The variant involves a change in polarity from a polar uncharged to a non-polar hydrophobic amino acid. The rarity of the variant, its position within the fibronectin type III (FN III) functional domain (23) and the high degree of sequence conservation of the amino acid residue across species, increase the likelihood of its pathogenicity (Table 1).

The c.1744_1751delinsT non-sense truncating variant also present in the first patient has been previously described in a case of autosomal recessive Leber Congenital Amaurosis in Turkey (24). It is predicted to cause nonsense-mediated mRNA decay or premature termination of translation producing a shorter protein missing the kinase domain. Consequently, this mutation is predicted to be an autosomal recessive pathogenic variant with deleterious effect on protein structure and function (7), which suggests this is a functional null allele. Therefore, in the presence of a severe truncating mutation and a moderately severe RP phenotype, we hypothesise that c.1133C>T is possibly a hypomorphic variant that might account for reduced protein function.

The second patient was homozygous for the c.2163T>A, p.His721Gln variant. This has been described in a female of Albanian descent by Audo *et al*. (7) and in a homozygous 28-year-old male by Colombo *et al* (10). The phenotype presented by Audo *et al* is similar to that of the case we report here, mindful of differences in severity due to age at evaluation (26 years versus 43 years). The variant results in the substitution of a histidine residue at position 721 (polar, positively charged) to glutamine (polar, uncharged) within the highly conserved intracellular tyrosine kinase domain (23). The rarity of the variant, conservation of the residue across species, presence in a functional domain and *in silico* prediction tools support the likelihood that this variant is indeed pathogenic for RP and may represent a founder mutation from Albania or the surrounding region (Table 1).

The third case was heterozygous for the c.1866G>C, p.Lys622Asn missense variant. This has been described once in the literature by Ellingford *et al* (8) in a homozygous patient with a reported RP/rod-cone dystrophy phenotype from a British cohort. The variant is predicted to be deleterious by way of substitution of a basic lysine residue by a polar uncharged asparagine residue within the highly conserved intracellular tyrosine kinase domain (23). Our report adds valuable phenotypic and clinical data to characterise this variant and further the evidence regarding its possible pathogenicity.

In recent years, remarkable advances in the field of ocular gene therapy have generated promising therapeutic opportunities for inherited retinal dystrophies (25–28). *MERTK* has been the object of investigation for treatment strategies including adeno-associated viral (AAV)-mediated retinal gene therapy. This was first investigated in animal models, which yielded successful results (29–31). Vollrath *et al* (25) found that subretinal administration of recombinant adenovirus encoding *MERTK* reversed the RPE phagocytosis defect and rescued photoreceptors from degeneration in juvenile rats, so that treated areas appeared near normal in animal models that already demonstrated significant pathology (31). In 2016, Ghazi *et al*. conducted a phase I clinical trial in a cohort of six patients with RP who exhibited biallelic mutations in *MERTK*. They established that AAV-mediated subretinal gene therapy had acceptable ocular and systemic safety profile, although the clinical benefit was variable and limited (32). *MERTK* continues to be a promising target for gene therapy for several reasons: 1) there are validated pre-clinical models available for vector validation; 2) it is expressed in the RPE, which can be targeted efficiently for gene transfer (33,34); 3) the *MERTK* cDNA sequence (3kb) is encodable within AAV (4.7kb) with sufficient space for the required transcriptional and regulatory elements (35).

A more recent alternative to gene therapy is the utilisation of RNA-programmable CRISPR-associated (CRISPR-Cas) nuclease technology, a powerful tool which allows precise and site-specific modifications of genomic DNA. Base-editing tools consist of catalytically inactivated Cas-nucleases programmed to localize target DNA loci, paired with a base-modification enzyme (36). They permit the direct and irreversible conversion of a specific target DNA base, theoretically enabling the correction of point missense transition mutations in a programmable manner (37–39). The cytosine to thymine single base mutation in the c.1133C>T variant here described might therefore be suitable for targeting with adenine base editors to revert the mutated A•T base pair back to G•C on the reverse strand (39). Prime-editing—a complementary DNA-editing tool that utilises the CRISPR-Cas9 targeting specificity with an RNA template that is inserted following reverse transcription—may edit transversion mutations (40). Therefore, the c.2163T>A and c.1866 G>C variants are theoretically correctable using this technology, or alternatively, by gene replacement therapy.

The identification of missense mutations in *MERTK* is important to better describe the spectrum of clinical phenotypes, to build the evidence base for pathogenicity of the variant and to identify potential candidates who may benefit from inclusion in clinical trials for retinal gene therapy. Base-editing and prime-editing tools are powerful technologies in which editing of pathogenic missense variants is possible.

## Supplementary Material

Supplemental Material
