# Supplementary material for: MERTK missense variants in three patients with retinitis pigmentosa
Source: Ophthalmic Genet. 2022 Aug 29;44(1):74–82. doi: 10.1080/13816810.2022.2113541 (PMC9615558; doi:10.1080/13816810.2022.2113541)
Supplement: Supplemental Material [file IOPG_A_2113541_SM0092.pdf]

Supplements Table

Variant	Protein change	ClinVar data	ACMG/AMP classification	PolyPhen-2	SIFT	Mutation Taster prediction	Grantham score	Align-GVGD Class
c.1133C>T	Thr378Met	Uncertain significance	Uncertain pathogenicity (PM2, PP3)	Score = 1 Probably damaging	0.00 (affect protein function)	Disease causing	81 – moderately conservative	C65 - most likely to interfere with function
c.2163T>A	His721Gln	Conflicting interpretations of pathogenicity (retinal dystrophy)	Uncertain pathogenicity (PM2, PP3)	Score = 1 Probably damaging	0.00 (affect protein function)	Disease causing	24 – conservative	C15 – second least likely to interfere with function
c.1866G>C	Lys622Asn	Uncertain significance (retinal dystrophy)	Uncertain pathogenicity (PM2, PP3, PM3_Moderate)	Score=1 Probably damaging	0.00 (affect protein function)	Disease causing	94 – moderately conservative	C65 – most likely to interfere with function
